# Hancock County Medical Association

**Published:** 1852-11

**Authors:** Thos. L. Barnes, Daniel Pierson

**Affiliations:** President pro tem.; Secretary pro tem.


*Hancock Co. Medical Association.*

At a meeting of a number of physicians, held in pursuance to previous notice, at Carthage, Hancock Co., Ill., Oct. 6th, 1852, for the purpose of organizing a County Medical Society, there were present: Drs. Thomas L. Barnes, Geo. W. Hall, and John Mack, of Carthage; Drs. Charles Coolidge, and H. Judd, of Warsaw; Dr. S. T. Ferris, of Fountain Green; and Dr. Daniel Pierson, of Augusta.

Dr. T. L. Barnes was called to the Chair, and Dr. D. Pierson appointed Secretary pro tem.

The object of the meeting was stated by Dr. Coolidge. On motion of Dr. Hall it was unanimously

Resolved, That we now proceed to organize a County Medical Society.

On motion of Dr. Coolidge, a Committee of two was appointed, by the Chair, to report a Constitution.

Chair appointed Drs. Coolidge and Hall, said Committee.

The Committee reported a Constitution, which, on motion, was read and adopted, article by article.

After which, on motion of Dr. Judd, it was

Resolved, That we now proceed to the election of officers. On the first ballot, Dr. Coolidge was elected President. Dr. Ferris was elected Vice-President. On motion of Dr. Barnes, Dr. Daniel Pierson was appointed Secretary. On motion of Dr. Judd, Dr. T. L. Barnes was elected Treasurer.

On motion of Dr. Judd, it was

Resolved, That a Committee of three be appointed by the Chair, to draw up Bye Laws, and a Code of Ethics, to be presented at the next meeting. On motion of Dr. Hall, it was

Resolved, That the Secretary be requested to furnish a copy of the minutes of this meeting, to the North-Western Medical and Surgical Journal, and to the papers at Warsaw, for publication.—On motion of Dr. Barnes, it was

Resolved, That when we adjourn, it be to meet at Carthage, on the third Monday in January, at 1 o'clock P. M.

On motion, adjourned.

THOS. L. BARNES, President pro tem.

DANIEL PIERSON, Secretary pro tem.

